# Lesions of “uncertain malignant potential” in the breast (B3) identified with mammography screening

**DOI:** 10.1186/s12885-018-4742-6

**Published:** 2018-08-16

**Authors:** Christiane Richter-Ehrenstein, Katharina Maak, Sonja Röger, Tilman Ehrenstein

**Affiliations:** 1 Department of Gynaecology and Obstetrics, Breast Unit, Klinikum Frankfurt (Oder), Müllroser Chaussee 7, 15236 Frankfurt (Oder), Germany; 2 Mammography Screening Unit Brandenburg Sued, Thiemstrasse 112, 03050 Cottbus, Germany; 3 Mammography Screening Unit Brandenburg Nord, Breitscheidstr.52, 16321 Bernau, Germany

**Keywords:** Breast neoplasm, Needle core biopsy, Positive predictive value, B3, Mammography screening

## Abstract

**Background:**

Core needle biopsy (CNB) is a standard diagnostic procedure in the setting of breast cancer screening. However, CNB may result in the borderline diagnoses of lesion of uncertain malignant potential (B3). The aim of this study was to access the outcome of lesions diagnosed as B3 category in a large series of screen-detected cases to evaluate the rates of malignancy for the different histological subtypes.

**Methods:**

We identified all CNBs over a six-year period (2009-2015) in a breast cancer screening unit in Germany. A total of 8.388 CNB’s were performed for screen detected breast lesions. B3 diagnosis comprised 4.5% (376/8.388). Of the 376 patients who were diagnosed as B3, 299 underwent subsequent excision biopsy with final excision histology.

**Results:**

Out of 376 patients diagnosed with B3 lesions, the prevalence of different histopathology showed 161 (42.8%) patients with atypical ductal hyperplasia (ADH), 98 (26.1%) with flat epithelial atypia (FEA), 50 women (13.3%) showed lobular neoplasia (LN), in 40 (10.6%) patients papillary findings and in 27 patients (7.2%) a radial scar complex. Final excision histology was benign in 74% (221/299) and malignant in 26% (78/299) of the patients. Lesion specific positive predictive values (PPV) for a subsequent diagnosis of in situ or invasive carcinoma were as follows: ADH 40%, FEA 20.5%, papillary lesion 13.5%, radial scar 16.6%, LN 0%.

**Conclusion:**

Our results show that approximately one-third of core needle biopsies of screen detected breast lesions classified as B3 are premalignant or malignant on excision.

Lesions of uncertain malignant potential of the breast (B3) are heterogeneous in respect to risk of malignancy.

## Background

The B classification [1] was proposed by the UK Breast screening program as a way of recognizing that between benign breast lesions (B2) and malignant lesions (B5) on core needle biopsy (CNB) lay a small group of lesions whose malignant potential could not be adequately ascertained by CNB alone (B3). These heterogeneous groups of lesions are of “uncertain malignant potential” and include various entities such as atypical ductal hyperplasia (ADH), flat epithelial atypia (FEA), lobular neoplasia (LN), papillary lesions and radial scars [1, 2]. Incidences for B3 lesions varies between the setting of breast cancer screening detected lesions and symptomatic detected lesions from 3% up to 17% with a tendency to higher rates in the screening detected group [3–8]. Despite the fact of ongoing research of risk tailoring of the different B3 entities most cases progress to surgical intervention in order to establish an excision histology diagnosis [9–12]. This has significant implications particularly in screen-detected breast lesions in which final benign diagnosis after surgical intervention is a drawback of mammographic screening. Recent recommendation support a consensus on which lesions may not require excision in particular circumstances [13]. We aimed to access the outcome of screen-detected lesions diagnosed as B3 category on routine practice in a large series of cases to determine the corresponding rates of malignancy for the various lesions. We also sought to determine a subgroup in which surgical interventions might not be appropriate in order to tailor therapy in this subgroup of women.

## Methods

We conducted a retrospective study of all women aged 50-69 who attended the German Mammography Program in the Screening Unit of Brandenburg/Sued between 2009-2015. Two - view digital screening mammograms were obtained from every participating woman. Each mammogram was reviewed by two independent specialized breast radiologists. For all abnormal findings a consensus discussion including a third reader was mandatory. Ultrasound as well as compressing views were used for recall patients before performing CNB. The need for needle core biopsy (CNB) was usually indicated after the finding of a mammographic abnormality that was not definitively benign. The CBN’s were performed using either ultrasound -guided core biopsies (14G) or stereotactic vacuum-assisted biopsies (11G). For US guided core biopsy 3-5 specimens were obtained, in case of a vacuum- assisted biopsy 12-16 specimen were achieved. According to the UK B-coding guidelines the results of the biopsies were classified as B1 to B5 -lesions (Table 1). In a multidisciplinary team approach all patients with a B3- lesion on CNB were discussed with an attending breast radiologist, pathologist and gynaecologist. The study was approved by the Ethics Commitee of the Brandenburgische Ärztekammer. Data collected included date of core biopsy, surgical procedure, CNB diagnosis (including lobular neoplasia (LN), atypical ductal hyperplasia (ADH), flat epithelial atypia (FEA) papillary lesion, radial scar) and final excision histological diagnosis. The postsurgical histopathological results were compared with the primary histopathological results of the core biopsy and the positive predictive value (PPV) for the detection of a premalignant or malignant lesion was calculated. PPV was determined as follows: PPV= (number of malignant cases/total number of subjects) x 100%.

**Table 1:** Reporting categories of Needle Biopsies

Category	Terminology	Meaning
B1	Normal tissue or uninterpretable	Normal or unsatisfactory biopsy
B2	Benign	Usual ductal hyperplasia, sclerosing adenosis, fibroadenoma, involutionary calcification, periductal mastitis, hamartoma
B3	Benign but of uncertain biological potential	Papillomas, radial scars/complex sclerosing lesions, lobular intraepthithelial neoplasia, atypical epithelial proliferation of ductal type, phylloides tumor
B4	Suspicious	Changes suggestive of in situ or invasive malignancy
B5	Malignant	Unequivocal malignant process
	a) in situ	
	b) invasive	
	c) uncertain whether in situ or invasive	
	d) other malignancies	

## Results

Between the years 2009-2015, a total of 8.388 CNB’s were performed for screen detected breast lesions. The lesion type depicted on mammography showed microcalcifications in 48% of cases, mass or density in 32% and architectural distortion in 20% of cases. Ultrasound -guided core biopsies (14G) were performed in 45% of cases for sonographically visible lesions, in 55% of CNB stereotactic vacuum-assisted biopsies (11G) were performed for lesions invisible for US or for the evaluation of microcalcifications. There was an association between the radiological finding and the upgrade rate in the final histology. Microcalcification was more likely to be associated with malignancy compared to other radiological features as architectural distortion or mass. In the group of stereotactic vacuum-assisted biopsies ADH as biopsy result was significantly more frequent than in the US-guided core biopsy group. B3 diagnosis comprised 4.5% (376/8.388). Of the 376 patients who were diagnosed as B3, data of 299 patients who underwent subsequent excision biopsy and final excision histology were available for review. Out of 376 patients diagnosed as B3, the prevalence of the different histopathological findings showed 161 (42.8%) patients with ADH, 98 (26.1%) patients with FEA, 50 women (13.3%) showed LN, in 40 (10.6%) patients papillary findings and in 27 (7.2%) subjects the histology of a radial scar complex. The group of 77 patients without excision biopsy results (57 patients denied further surgical intervention, 20 patients were lost in follow up) consisted of the following CNB diagnoses which showed a fairly similar distribution of B3 lesion types to the study population.: 26 patients with ADH, 25 patients with FEA, 3 papillary lesions, 3 radial scar complex and 20 patients with lobular neoplasia. Table 2 summarizes the distribution of all available CNB biopsies with consecutive excision histology outcomes (299 cases) for B3 core needle histology, overall and for each subcategory of B3 the category –specific Positive Predictive Value (PPV). Final excision histology was benign in 74% (221/299) and malignant in 26% (78/299) of the patients (48 ductal carcinoma in situ and 30 invasive carcinoma). Lesion specific PPV’s were as follows: ADH 40%, FEA 20.5%, papillary lesion 13.5%, radial scar 16.6%, LN 0%.

**Table 2:** Lesion of uncertain malignant potential (B3) on CBN with excision histology outcome for different B3 lesions and associated PPV for breast malignancy

Category of B3 diagnosis (CNB) with excision histology (number of cases)	Final excision diagnosis			
	Benign	Malignant in situ no. (%)	Malignant invasive no. (%)	PPV (%) excision histology
FEA (73)	58	11 (15.1)	4 (5.4)	20,5 (15/73)
ADH (135)	81	32 (23.7)	22 (16.3)	40 (54/135)
Papillary lesion (37)	32	2 (5.4)	3 (8.1)	13.5 (5/37)
Radial scar (24)	20	3 (12.5)	1 (4.1)	16.6 (4/24)
LN (30)	30	0	0	0 (0/30)
Total (299)	221	48 (16.1)	30 (10)	26,1 (78/299)

*FEA* flat epithelial atypia, *ADH* atypical ductal hyperplasia, *LN* lobular neoplasia, *CBN* core needle biopsy, *PPV* positive predictive value

## Discussion

In breast cancer screening the main aims are early detection and management of breast cancer in order to reduce mortality from breast cancer. At the same time the program tries to avoid unnecessary surgery for benign disease in screened asymptomatic women. So therefore the management of lesions as of uncertain potential (B3) by needle core biopsy is essential for the success of breast cancer screening.

In our large study cohort about 4.5% of all consecutive CNBs performed between 2009-2015 have been reported as B3 (lesions of uncertain malignant potential). In a screening cohort published by El-Sayed et al [14] they reported 5% as B3 lesion what seemed to be fairly low in comparison to other groups. Data from Germany published in 2011 reported 15% of B3 lesion in a screening cohort [15].

Overall, 26% (78/299) of borderline lesions on CNB in this study proved to be DCIS or invasive carcinoma; however the PPV for each subcategory within B3 varied substantially (Table 2). Lee et al [8] provide data on histological outcomes for the B3 category with an overall PPV of 26%, Houssami et al [5] reported a slightly higher PPV of 35.1% and in a large cohort study as Weigel et al [15] reported from a German screening group overall PPV rates of 28%.

The most frequent lesion in our series is ADH and its frequency within the B3 category is similar to that reported by Lee, Houssami and others. The PPV for ADH (40%) emphasizes that the standard of care for lesions yielding ADH on CBN should be excision biopsy. Additionally newly published research data showed that atypical ductal hyperplasia has shown to confer a relative risk for future breast cancer of 4 [10]. The differentiation of ADH and low grade ductal carcinoma in situ is a question of the extent of the lesion more than of histological features which show great similarities. More recent data on absolute risk from the Nashville Breast Cohort confirm the cumulative high risk of breast cancer among women with atypical hyperplasia to be 30% 25 years after the diagnosis of atypical hyperplasia by CNB [10].

The second frequent lesion in our series is a flat epithelial atypia (FEA) with a percentage of 26,1% in our CNB screening cohort. In comparison to data published by authors of the UK breast cancer screening [3, 4] who found a significant lower percentage of FEA with less than 10% of the detected lesions our results are in concordance with published work mostly done outside the UK breast cancer screening program [16, 17]. This may in fact represent the question of histopathological diagnosis of B3 lesions with the introduction of the term of AIEP (atypical intraductal epithelial proliferations) and the known resulting variability in the literature. In recent years a number of studies discussed a potential relationship between FEA, atypical hyperplasia, and low-grade carcinoma [18]. The PPV for FEA in our study was 20.5% with 11 patients showing in situ lesions and 4 (total 15/73) diagnosed as cancer on excision biopsy. Data from a recent meta-analysis, carcinoma was present in the excisional biopsies of 13-67% of cases of FEA diagnosed on CNB [19]. This analysis included studies without radiological-pathological correlation. Keeping in mind that breast cancer development is a question of time as we discussed the results of the Nashville Breast Cohort, observation may be an acceptable option as pointed out from the World Health Organization Working Group [20]. In our view it should be restricted to very small lesions and complete removal of imaging abnormalities.

Concerning the PPV for papillary lesions (13.5 % for papillary lesion in our series) in which 3 out of 5 cases showed atypia on CNB with a final malignant histology which is similar to a recent meta-analysis [21], surgical management for papillary lesions require a distinct pathological report with respect to the question of atypia.

Patients with atypical papillary lesions or papillary carcinoma on CNB should be routinely referred for surgical consultation and excision. Without findings of atypia and after a sufficient representative biopsy and radiological concordance, excision biopsy might be omitted [22, 23].

Our results showed a PPV for radial scars of 16.6% with 3 cases of DCIS and one case of an invasive tumor which is in line with several studies correlating CNB diagnosis of radial scars with final histology after surgery. They have shown variable upgrade rates ranging from 0% to 40%. All 4 cases showing DCIS or a malignant histology on surgical excision were larger than 10mm. This is in accordance with data from Conclon et al who reported an overall upgrade rate of 7.5% for radial scars without atypia. In the majority of cases of upgrades in these series DCIS was diagnosed as well as that radial scars of <5mm were associated with a lower upgrade rate (≤2%) and may not require excision [24, 25].

Unlike the PPV for lobular neoplasia in our series (0%) which is not clearly understood several studies reported on high lesion specific risk of malignancy with rates from 8% up to 60,9% [5, 14, 26, 27]. LN is usually an incidental finding in the setting of mammography screening but in some cases it is associated with microcalcifications. A recent review by Calhoun BC and Collins LC discussed that the upgrade rate of LN is low as ≤2% especially in cases when LN is not in association with the radiological target lesion [13, 28, 29]. With regard to personal risk factors as a positive family history or others it should be emphasized that multidisciplinary meetings are warranted to decide in which patient observation may be appropriate [30, 31]. The National comprehensive cancer Network 2015 Guidelines recommended surgical excision for lobular neoplasia diagnosed on a CNB [30, 31].

In view of the existing literature our data clearly support the heterogeneous biology of B3 lesions diagnosed by CNB. The study is highly relevant for the work of the German mammography screening program as it is to our knowledge the largest study on this topic in Germany. Our study supports the strategy to define subgroups, in which surgical intervention might not be appropriate, but highlights the value of surgical treatment for distinct entities. Limitations of our study that had to be discussed are on one hand the relatively rare diagnosis of lobular neoplasia as well as the retrospective design of our study. Furthermore one has to consider the different use of diagnostic terms for identical pathological lesions that may hinder a clear comparison of pathological diagnosis over the recent years.

## Conclusion

Our results show that approximately one-third of core needle biopsies of screen detected breast lesions classified as B3 are premalignant or malignant on excision.

Lesions of uncertain malignant potential of the breast (B3) are heterogeneous in respect to risk of malignancy.

There is evidence that not all patients with B3 lesions need surgery but careful radiological-pathological correlation is the prerequisite for accurate treatment. It will be a critical role for multidisciplinary teams applying histological criteria and diagnostic terminology to guide risk stratification and appropriate patient management.

## Abbreviations

ADH: Atypical ductal hyperplasia
AIEP: Atypical intraductal epithelial proliferations
CNB: Core needle biopsy
DCIS: Ductal carcinoma in situ
FEA: Flat epithelial atypia
LN: Lobular neoplasia
PPV: Positive predictive value
